# Conducting trials on corticosteroid dosing for respiratory failure in the last paradise

**DOI:** 10.1186/s40560-018-0346-0

**Published:** 2018-11-20

**Authors:** Nobuaki Shime

**Affiliations:** Department of Emergency and Critical Care Medicine, Postgraduate School of Medical Science, Hiroshima University, 1-2-3 Kasumi, Minami-ku, Hiroshima, 734-8551 Japan

## Abstract

It is interesting to find that Japanese clinicians continue to hesitate to change their practice even after accumulating evidence for the inefficacy of high-dose corticosteroid for ARDS in the Letters to the Editor discussion. Given the widespread use of the therapy even for other categories of acute hypoxemic respiratory failure with diffuse alveolar damage represented by acute exacerbation of interstitial pneumonia, Japan is the last part of the world in which efficacy of corticosteroid dosing (including pulse therapy) is assessed in those patients if they wish to continue this trend.

## Text

I read with great interest the Letter to the Editor from Meduri et al. [1] for the English version of the “Clinical practice guidelines for the management of adult patients with ARDS” published recently in the *Journal of Intensive Care* [2] and the reply from the authors [1].

Inclusion of an old randomized controlled trial that investigated high dose of methylprednisolone therapy (120 mg/kg over 24 h) [3], which is termed “methylprednisolone pulse therapy,” was discussed. It appears that the practice and scientific arguments have been forgotten from Western countries owing to the negative trials conducted approximately two decades ago.

Interestingly, however, the Japanese guideline on acute respiratory distress syndrome (ARDS) management is unable to ignore the therapy, and in fact, Japanese clinical practice appears to adhere that according to a nationwide survey [4]. It would be interesting to know why Japanese clinicians continue to hesitate to change their practice even after a recent publication from a Japanese hospital showing the potential harm of pulse therapy, which was found to be significantly associated with higher mortality and a reduction in the number of ventilator-free days [5].

Moreover, routine use of pulse therapy for acute exacerbation of interstitial pneumonia continues in Japan without robust evidence for its use. No studies have shown clinical benefit in using high-dose corticosteroids in patients with acute exacerbation of idiopathic pulmonary fibrosis (a more severe form of interstitial pneumonia) [6, 7]. Although acute exacerbation of interstitial pneumonia is a distinct disease category and is not necessarily associated with diffuse alveolar damage, there is undoubtedly an option for extrapolating the potential beneficial effect and safety of lower-dose corticosteroid therapy, as suggested by Meduri et al., [1] for the difficult-to-treat disease.

Japan is probably the last Galapagos paradise in the world in which assessment of the efficacy of corticosteroid dosing (including pulse therapy) in patients with various categories of acute hypoxemic respiratory failure with diffused alveolar damage (such as ARDS or acute exacerbation of interstitial pneumonia) can be done. Future clinical trials by Japanese clinicians should be conducted if they continue staying in the paradise.
